# Physio-Metabolic Mechanisms Behind Postharvest Quality Deterioration in Broccoli (*Brassica oleracea* var. Italica) and Swiss Chard (*Beta vulgaris* L. var. Cicla): A Review

**DOI:** 10.3390/plants13223174

**Published:** 2024-11-12

**Authors:** Sabelo Shezi, Mduduzi E. K. Ngcobo, Nokuthula Khanyile, Khayelihle Ncama

**Affiliations:** 1 School of Agricultural Sciences, Faculty of Agriculture and Natural Sciences, University of Mpumalanga, Mbombela 1200, South Africa; mduduzie.ngcobo@gmail.com; 2 School of Chemical and Physical Sciences, Faculty of Agriculture and Natural Sciences, University of Mpumalanga, Mbombela 1200, South Africa; khanyile.peaceful@gmail.com; 3 Department of Horticulture, Durban University of Technology, Durban 4001, South Africa; khae.ncama@gmail.com

**Keywords:** fresh produce, leafy vegetables, postharvest quality, marketability, shelf life

## Abstract

Leafy vegetables are among the potential foods that can combat food insecurity in developing countries. Their major drawback is a short shelf life, which limits their supply chain and is commonly associated with their high metabolic activities. Leafy vegetables have a high water content, which determines their freshness. Moisture loss through respiration and transpiration at postharvest storage is one quality attribute that leads to rapid quality deterioration. Little has been carried out in studying the mechanisms associated with the quality deterioration of leafy vegetables; however, understanding these mechanisms may aid in developing effective preservation measures. Furthermore, recent literature reviews that focus on discussing the mechanisms that lead to quality loss in leafy vegetables are scarce. The current paper aims to review the physiological and biochemical processes associated with quality deterioration in leafy vegetables. The respiration, ethylene production, moisture loss, colour, and texture are highly associated with the quality deterioration of fresh produce and, thus will be discussed critically in selected leafy vegetables, namely: broccoli and Swiss chard. The findings from this review indicate that the quality deterioration in leafy vegetables is primarily enzymatic. Understanding the mechanisms of quality deterioration involves identifying the specific enzymes responsible for each metabolic process and examining the internal and external factors that influence enzyme activities.

## 1. Introduction

Leafy vegetables form a very important component of the human diet [1], as they consist of antioxidants and other metabolites while being a reliable source of vitamins and minerals. Consumption of various leafy vegetables is associated with attaining a higher content of various vitamins, including vitamins C, A, E, B1, B6, and B9. Leafy vegetables are also rich in dietary fibre, carotenoids, proteins, and several minerals [2]. As a result, consuming leafy vegetables has been linked to several health benefits, including controlling blood sugar, boosting the immune system, reducing blood pressure, promoting bone growth strength, and improving brain function [2].

The availability of leafy greens in the fresh produce market is limited because they are highly perishable [3]. Leafy vegetables are characterized by higher metabolic processes that accelerate quality deterioration [4]. They have higher respiration, ethylene production, and evapotranspiration rates, which result in several physiological and biochemical responses including chlorophyll degradation, loss of texture, moisture loss, as well as conversion of organic acids to sugars. Higher metabolic processes have been reported to reduce the quality of fresh produce, such as fruits and non-leafy vegetables [5]. The metabolism is even higher in leafy vegetables because of the higher surface area-to-volume ratio.

Understanding the mechanisms behind quality deterioration in leafy vegetables can aid in developing appropriate preservation measures. The current paper aims to review the physiological and biochemical processes associated with quality deterioration in leafy vegetables. Therefore, respiration, ethylene production, moisture loss, colour, and texture will be critically discussed in broccoli, and Swiss chard. Furthermore, biochemical processes like loss of ascorbic acid, chlorophyll, carotenoids, phenolic compounds, total antioxidants, and the accumulation of enzymes like peroxidase (POD), polyphenol oxidase (PPO) and phenylalanine ammonia (PAL) will be discussed critically. This study attempts to explain the general mechanism by which leafy vegetables lose quality and deteriorate. Expanding on a few selected vegetables was conducted based on them being the most common and significantly contributing to the vegetable industry globally. An understanding of the mechanism of quality deterioration will improve the postharvest handling of these crops globally and improve the overall revenue of vegetables. The previous literature reviews are acknowledged and presented in Table 1 but none of them critically discuss the content that is covered in the current review paper. The objectives and contributions of the reviews were summarized for ease of understanding.

## 2. Materials and Methods

All the materials used to develop this review paper were obtained from the scientific databases available online, including Google Scholar, Research gate, Science Direct, Web of Science, and Scopus. The key subtopics discussed in this paper review were searched based on various keywords including “leafy vegetables”, “senescence of leafy vegetables”, and “postharvest physiology of leafy vegetables”. Almost all the relevant literature was obtained by using the above keywords; however, for more advanced biochemical processes involved in vegetables shelf life the specific phrases such as “moisture loss”, “ethylene production”, “respiration”, and “texture” were used. Only the newly published paper reviews and research articles were targeted for developing this paper review; however, the timeframe restriction was removed due to the scarcity of the information on the subject. The most recent articles were of the priority in most discussions to ensure the relevance of the document. Approximately 75 research articles, book chapters, and paper reviews were used to develop this paper review.

## 3. Physiological and Biochemical Changes Associated with Quality Deterioration

The postharvest quality and shelf life of leafy vegetables are regulated by a number of physiological and biochemical processes that lead to quality deterioration [14]. These include the rate of respiration, ethylene production, moisture loss, chlorophyll degradation, and loss of texture [14,15]. Once started, each of these processes cannot be stopped, but their rate can only be retarded after the leafy vegetables have been harvested. These are genetically programmed and highly coordinated, as a result, they are species dependent. These processes influence each other and their occurrence at higher rates collaborate, resulting in hastened senescence. They are all influenced by temperature, relative humidity, and atmospheric gas composition during the storage of fresh produce.

### 3.1. Respiration

Respiration is a catabolic process in which accumulated carbohydrates are converted into usable forms in the presence of oxygen. This process occurs in fruits and vegetables, allowing fresh produce to convert the energy accumulated as photosynthates to be utilized to survive independently [16]. Although the respiration mechanism is the same in fruits and vegetables, the rate differs, and it is higher in leafy vegetables [10]. The reason is that initially, the leaves act as sink organs during early development stages and then become the source as they mature and engage in photosynthesis [17]. Leafy vegetables primarily function as sources of photosynthates due to their active role in photosynthesis [18]. Consequently, their morphological structure is adapted to being sources rather than storage organs, which is unlike fruits. The leafy vegetables have a huge surface area to volume ratio, which makes them less conservative towards accumulated carbohydrate reserves [19]. For postharvest survival, leafy vegetables need to function as sink organs and store sufficient carbohydrates. These are then needed as energy sources to sustain other biochemical processes after harvest. Inadequate carbohydrate conservation leads to high perishability and shorter postharvest life [19,20]. Additionally, leafy vegetables contain approximately 80% water, and their shape is maintained by water (hydro-skeleton) [21]. Upon harvest, they lose water rapidly, contributing to quality deterioration.

#### 3.1.1. Broccoli

Respiration is an important metabolic process that involves the breakdown of organic compounds, such as sugars and starch to produce energy [22]. This process is essential for maintaining various physiological activities and supporting the growth and development of broccoli. Nonetheless, respiration also contributes to the deterioration of the quality of broccoli over time, especially when it is not properly controlled [23]. Understanding the role of respiration in post-harvest physiology assists with implementing efficient storage methods and preservation strategies to maintain the quality and freshness of broccoli.

During respiration, stored carbohydrates such as starch and sugars are consumed to release energy for cellular activities, which reduces the number of available nutrients [23]. Prolonged storage under oxygen-rich conditions can lead to a decline in the overall nutritional value of broccoli since oxygen encourages the breakdown of nutrients such as vitamin C [22]. Additionally, respiration contributes to the accumulation of by-products such as carbon dioxide; high levels of carbon dioxide in storage can create a conducive environment for microorganisms, causing spoilage and decay [22]. Respiration is directly proportional to high temperatures, which results in high respiration, leading to faster deterioration [23]. High temperatures speed up metabolic and enzymatic activities, accelerating the breakdown of cellular components and promoting senescence.

#### 3.1.2. Swiss Chard

According to Berna [24], fresh vegetables continue to respire after harvest; therefore, poor postharvest handling and storage can quickly cause damage and reduce the crops’ nutritional and sensory value. The chemical process known as respiration is how fruits and vegetables break down sugars in the presence of oxygen to form carbon dioxide, water, and energy in the form of adenosine triphosphate (ATP) [25]. The heat produced during respiration causes a commodity’s temperature to rise. The respiratory activity of leafy vegetables affects how long they can be stored. A commodity can have its storage life extended by delaying senescence and reducing respiration when it is stored at a low temperature [26].

Lowering the rate of respiration can also be accomplished by carefully managing the concentrations of carbon dioxide and oxygen in the Swiss chard leaves. Swiss chard leaves respiration rate increases and then gradually decreases over time; this is because after harvesting, the plant tissues continue to respire. If the harvested Swiss chard leaves are exposed to high temperatures and high humidity, the respiration rate will increase, resulting in faster depletion of stored carbohydrates and nutrients, leading to the decline in the overall quality of the produce [27]. These findings suggest that the successful management of respiration through controlling temperature and oxygen concentration could result in maintaining the quality of Swiss chard. Therefore, developing technologies such as precooling, controlled atmosphere, modified atmosphere packaging, and cold storage that serve this role is of high importance in the fresh produce industry.

#### 3.1.3. Respiration in Other Leafy Vegetables

The respiration rate has been reported to start higher at harvest and continuously decrease during lettuce storage [28]. The higher respiration rate at harvest is linked to the responsive shock of the lettuce heads due to being detached from the roots at harvest [29]. The abnormal metabolic response at harvest serves as an adaptation mechanism towards the life after harvest [30]. Elevated respiration is commanded by the demand for energy in the form of ATP required by the leaf tissues to sustain themselves since they are no longer photosynthesizing [31]. A continuous reduction in the respiration rate of lettuce is associated with the adaptation of tissues to life after harvest [32]. Woltering and Witkowska [32] further reported that the continuous reduction in the respiration rate in lettuce after harvest is linked to the reduction in the sugars and oxygen as substrates of respiration. Luna et al. [33] reported that temperature serves as a rate determinant for the rate of respiration in lettuce and other greens, as it speeds up the metabolic processes. Therefore, the current findings reveal that the rate of respiration starts higher in lettuce and continuously decreases, and it is influenced by the temperature. Oxygen also serves as a key substrate in the respiration reaction; therefore, controlling oxygen and temperature surrounding fresh produce can preserve the quality and extend the shelf life of leafy greens. New technologies that limit the exchange of gasses into and out of the fresh produce are potential solutions when it comes to regulating respiration in leafy greens. Low non-freezing temperatures slow down the metabolic processes and therefore, have a huge potential to preserve the quality of fresh greens; however, prolonged storage reduces quality. Furthermore, optimum management practices that will maximize the amount of carbohydrates and freshness at harvest also serve a key role in the postharvest quality of fresh produce.

### 3.2. Ethylene Production

**Figure 1:**
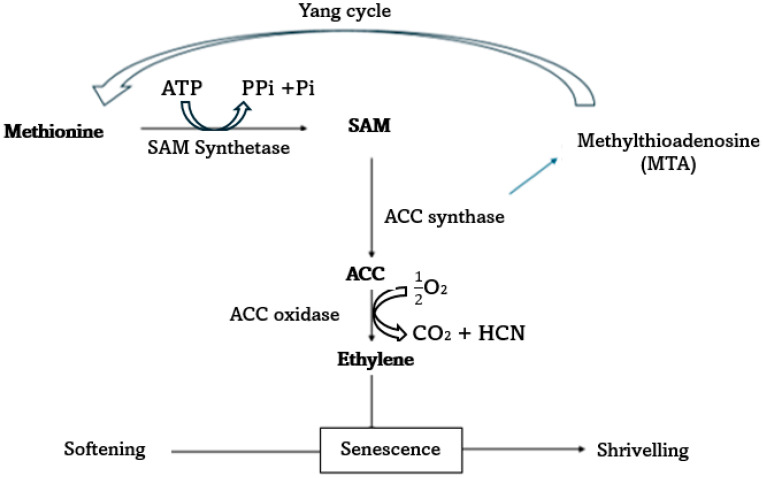
Mechanism for ethylene production in fruits and vegetables, a modified diagram [40].

Ethylene production is an autocatalytic process that emanates from its biosynthesis (Figure 1). The biosynthesis pathway of ethylene involves methionine, which serves as a major precursor to ethylene. The role of adenosine triphosphate (ATP) is to empower the reaction that involves the dissociation of methionine to form S-adenosyl methionine (S-AdoMet.). This reaction is catalyzed by the enzyme called S-adenosylmethionine (SAM) synthetase [34]. S-adenosyl-L-methionine (SAM) and 1-aminocyclopropane-1-carboxylic acid (ACC) are key intermediates in the biosynthesis of ethylene [34]. In this pathway, ACC synthase (ACS) converts S-adenosyl methionine to ACC, and ACC oxidase (ACO) then converts ACC to ethylene [35]. The role of oxygen is to facilitate the activity of an enzyme ACO which catalyzes the oxidation of ACC to form ethylene. Therefore, oxygen serves as a substrate for oxidation [34]. The biosynthesis pathway of ethylene is presented in Figure 1. This process occurs in all fresh produce, including fruits, vegetables, and ornamentals. The rate of occurrence varies with the species, as it serves as a ripening hormone in climacteric fruits. In leafy vegetables, ethylene production occurs constantly, and it stimulates senescence when produced excessively.

In the study by Diaz et al. [36], selenium (Se) reduced ethylene production in chicory and lettuce. The mechanism linked to this is the ability of Se in plants to be converted to Se-methionine, which reduces free methionine that serves as an initial substrate of ethylene biosynthesis [36,37]. Ahlawat et al. [38] recently reported that ethylene production is linked with senescence-associated genes (SAGs) in Brassicaceae species like cabbage, kale, and broccoli. In this study, the named leafy vegetables were stored at room temperature (25 °C) and at 4 °C. At room temperature, the expression of the SAG genes like *ORE15, SAG12*, and *NAC29* increased, while the expression of *ORE15* and *SAG12* was reduced at lower a temperature (4 °C). The findings from this study revealed that higher temperature promotes gene expression. This study further revealed that the low temperatures selectively reduce the expression of SAGs and the ethylene receptors.

Cutting leafy vegetables triggers the production of ethylene by the plant tissues, which then induces accelerated senescence that leads to quality deterioration in leafy vegetables. Guan et al. [39] reported that the production of phytohormones like ethylene, jasmonic, and salicylic acid is induced by wounding. Wounding activates the biosynthesis of the secondary metabolites that naturally serve as protective agents against oxidative stress. Furthermore, wounding provides additional pores for the exchange of gasses into and out of the plant tissues and hence exacerbates the metabolic rates associated with quality deterioration. Therefore, it is evidenced from the studies above that the mechanism of ethylene production is linked to the availability of methionine, the precursor of ethylene production, and the senescence-associated genes (SAGs). They further revealed that ethylene production is enhanced by higher temperatures and wounding.

#### 3.2.1. Broccoli

Ethylene plays a crucial role in the post-harvest physiology of broccoli, as it influences the ripening and senescence processes [22]. After harvesting, broccoli continues to produce ethylene depending on factors such as temperature and storage conditions. One of the most prominent effects of ethylene in broccoli is its involvement in the conversion of starches into sugars, contributing to flavour enhancement [41]. Furthermore, broccoli exposure to ethylene causes the production of volatile organic compounds, contributing to the development of the characteristic aroma and flavour profiles [41].

While ethylene plays a significant role in enhancing certain quality traits of broccoli, excessive exposure speeds up the rate of deterioration during post-harvest handling. According to Cai et al. [41], ethylene induces senescence, compromising the nutritional value of broccoli by promoting the degradation of nutrients. The breakdown of essential vitamins and antioxidants occurs in the presence of ethylene, resulting in the decline of the nutritional state of the commodity [41]. The health benefits associated with consuming broccoli will also diminish, reducing its overall market value. Ethylene is also associated with the loss of chlorophyll, which leads to the yellowing of the broccoli florets [22].

#### 3.2.2. Swiss Chard

All vegetables are non-climacteric, which means that they contain a constant amount of ethylene gas throughout their growing season compared to climacteric produce, which displays a peak in ethylene production during their ripening stage [42]. After harvesting Swiss chard leaves, the ethylene production continues, resulting in the leaves losing their green colour due to chlorophyll breakdown and making them turn yellow. Additionally, the senescence of vegetative issues will occur over time, resulting in the decaying of the produce [42]. Ensuring that the harvested Swiss chard leaves are kept in good condition can assist in reducing how quickly the tissue reacts to ethylene activity, thereby delaying spoilage and maintaining the quality of the produce.

### 3.3. Moisture Loss

**Figure 2:**
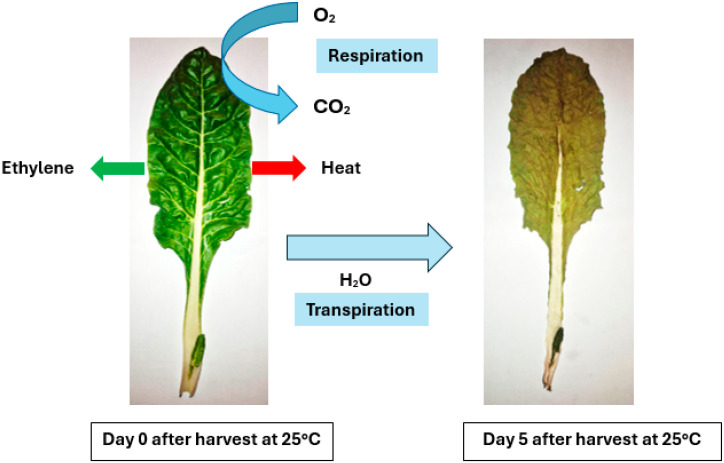
Swiss chard changes during 5 days of storage at 25 °C.

Moisture loss in fresh produce is primarily driven by transpiration, the process by which water is lost from the aerial parts of plant tissues, mainly through stomata (Figure 2) [43,44]. The water loss leads to dehydration and shrivelling of the plant tissues. Transpiration is influenced by external factors such as temperature, relative humidity, and atmospheric composition around the plant tissue [45]. Transpiration also causes moisture loss due to the water vapour pressure deficit between the leaf and the surrounding environment. A higher water potential in the leaf compared to the environment leads to osmotic diffusion of water from the leaf to the atmosphere [44]. Consequently, moisture loss increases under low humidity due to a greater water vapour pressure deficit, while moderate humidity reduces moisture loss, extending the shelf life of leafy vegetables [44]. Noteworthy, extremely high humidity can promote the rapid production of cells or indirectly reduce their quality by providing an ideal environment for fungal disease development.

#### 3.3.1. Broccoli

Broccoli contains a high percentage of water content, which contributes to the crispiness, juiciness, and freshness of the commodity [46]. As broccoli respires, it loses water through transpiration and respiration, leading to a reduction in mass and deterioration in quality [46].

Excessive water loss leads to wilting, shrivelling, and a decline in visual appearance. Maintaining low temperatures and high humidity can assist with minimizing water loss [47]. In an experiment by Makino and Amino [46], broccoli was stored at a temperature of 10 °C with 80% humidity, for a planned duration of 14 days. However, by day 8 of storage, there was shrinkage in the flower bud, the shrinkage was attributed to water loss.

Moisture loss occurs primarily through the evaporation of water from broccoli tissues during storage and handling, resulting in a change in appearance, texture, and nutritional content [46,47]. The primary consequence of mass loss is the loss of turgidity that results in wilting and softness of florets. As the water evaporates, cells become less turgor, causing them to become less crisp; loss of crispness affects the appearance making it less appealing in the eye of the consumer [46].

Wilting can accelerate quality deterioration by increasing its susceptibility to mechanical damage and microbial spoilage since weakened tissues are more susceptible to penetration by pathogens and bruising. Moreover, a study by Makino and Amino [46] shows that mass loss can lead to weight loss during storage; thus, affecting the economic viability of producers and distributors.

#### 3.3.2. Swiss Chard

According to Zhang and Xie [47], moisture loss is the cause of harvested green vegetables shrinking and losing weight/mass during storage. This is because most leafy vegetables have a high water content (85–95%), which increases their susceptibility to rapid water loss due to their higher surface area to volume ratio [48]. The loss of the moisture content in the leaves can result in wilting and loss of freshness and texture. This is mainly because after harvesting fresh greens, the water that is being lost through transpiration is no longer replenished [49,50]. Several studies on spinach revealed that a loss of moisture is associated with quality deterioration [47,48,49,50].

### 3.4. Colour

Colour is among the key quality attributes of leafy vegetables as it directly affects the visual appearance of fresh produce [51]. The visual appearance is the most important trait for attracting consumers to buy fresh produce under wholesale and retail conditions [52]. This trait is known to be strongly correlated with the organoleptic quality and nutritional content, so a poor relationship between these results in no repeated buys by customers [51]. Therefore, colour serves as the most important quality attribute in leafy vegetables, and it must be preserved until the produce is sold. The green colour, which occurs due to chlorophyll in leafy vegetables, is influenced by many internal and external factors [53]. A loss of green colour is a marker of quality deterioration in leafy greens, and it occurs due to chlorophyll and carotenoid degradation [6,54]. Discolouration in leafy greens is also caused by browning that is induced by the enzyme called polyphenol oxidase, which catalyzes the oxidative reactions of phenolic compounds to produce various polymerized products.

#### 3.4.1. Broccoli

Colour change is a noticeable aspect of post-harvest physiology, as it significantly affects the visual appeal and consumer acceptance of broccoli. The radiant green colour of fresh broccoli is due to the presence of chlorophyll, the pigment that is responsible for photosynthesis [55]. The presence of chlorophyll indicates that the broccoli is fresh and of good quality. However, this gradually declines after harvesting. More studies reporting colour change are presented in Table 2.

One of the colour changes observed in broccoli after harvest is the transition from vibrant green to a yellowish hue [56], and this is caused by chlorophyll degradation in fresh produce. The process of chlorophyll breakdown is influenced by factors such as exposure to light, high temperature, and ethylene. Exposure to ethylene gas, which is produced by broccoli itself and other surrounding produce, can accelerate chlorophyll degradation and colour changes [55].

In summary, the loss of green pigments, known as chlorophyll and the development of yellow pigments, known as carotenoids, indicates senescence and potential quality deterioration [56]. Managing factors such as ethylene exposure and storage conditions can assist in minimizing the rate of deterioration. Therefore, the adoption of ethylene and oxygen controlling technologies like modified atmospheric packaging (MAP) and controlled atmospheric storage could play a significant role in maintaining the quality of leafy greens.

#### 3.4.2. Swiss Chard

The most typical postharvest disorder that reduces the fresh-produce’s economic value is the colour changes. Most leafy vegetables are associated with green colour, which implies that the product is fresh and in good quality. In Swiss chard leaves, the leaves are green in colour; however, the characteristic colour of its petioles is classified as either white or red depending on the cultivar. Betacyanin, a substance closely linked to anthocyanin responsible for most of the red colours in plants, is primarily responsible for the red colour [57].

The colour change that occurs during the post-harvest period might be caused by ethylene, unfavourable storage conditions, mechanical damage, enzymatic abnormalities, or hormonal imbalances [51]. It is important to ensure that Swiss chard leaves are kept under dark storage conditions because exposing the produce to light conditions has hastened the leaves to turn yellow due to the degradation of chlorophyll [51]. This can result in postharvest losses because the colour will not meet the consumer’s satisfaction, resulting in decreased sales. Future research should focus on developing new technologies that limit mechanical injuries during handling because mechanical injuries hasten all physiological processes, leading to senescence.

### 3.5. Texture

The texture of leafy vegetables is regulated by the water content and the enzymatic activities [58]. At harvest, the cells in leafy vegetables are turgid and full of water, and this keeps the texture higher and at optimum quality. As the cells lose turgidity, they lose texture and shrivel. The loss of moisture and the movement of water in the leaves is regulated by mineral nutrients like potassium, and the regulation of the cell wall and membrane structure is controlled by calcium. The functioning of the membranes is controlled by the enzymatic activities. The nature of softening emanates from the dissolution of the middle lamellae between the cell membrane and the cell wall [59]. The enzymes involved in regulating the effective functioning of the membranes are polygalacturonase (PG) and pectin methyl esterase (PME). Polygalacturonase and PME are responsible for the degradation of pectin, which is associated with a loss of texture [60]. Therefore, the higher activities of these enzymes stimulate the loss of texture, characterized by shrivelling in leafy vegetables. As the cells lose water, they become flaccid or collapse.

#### 3.5.1. Broccoli

Texture is determined by the composition of cell wall components such as cellulose, hemicellulose, and pectin [61]. Post-harvest handling and storage conditions can impact these structural components leading to changes in texture over time. Freshly harvested broccoli displays firmness and crispiness, which is due to the intact cell water and high moisture content [61]. Yet over time, as broccoli experiences post-harvest metabolism, texture changes become evident.

One of the main factors causing textural changes is the enzymatic degradation of cell wall components. Enzymes such as cellulases and pectinases break down cellulose and pectin molecules, which weakens the structure of the cell walls and leads to softening of broccoli tissues [62]. As firmness decreases over time, appearance and consumer appeal diminish. Additionally, moisture loss results in loss of turgor pressure, leading to the softness of florets and stems. Future research should focus on developing new technologies and storage that will limit moisture loss and, hence, transpiration from tissues. These technologies will optimize the freshness, turgor, shininess, and crispness in leafy greens since moisture loss results in the loss of all these quality attributes.

#### 3.5.2. Swiss Chard

The structural, physiological, and biochemical properties of living cells and how they alter during the phases of development, maturation, and senescence determine the texture of vegetables [63]. As vegetables ripen, the cell wall breaks down, and pectin is also degraded. A net loss of galactose and arabinose, and maybe a reduction in the molecular weight distribution of hemicelluloses [64]. As a result, after harvesting the Swiss chard leaves, the turgor pressure of the cell wall decreases, resulting in leaves that are flabby, soft, spongy, or withered over time, provided that the produce is exposed to high temperatures [64]. This may also result in leaves that have a poor crispy and crunchy texture, which may result in losses because consumers associate the crispness and crunchiness of leaves with freshness and healthiness [65].

### 3.6. Antioxidant Activity Associated with Quality Deterioration in Leafy Vegetables

A study by Liao et al. [66] revealed that quality deterioration in leafy vegetables like spinach, cabbage, and leeks at 4 °C storage is associated with a loss of primarily chlorophyll content, then ascorbic acid and soluble proteins. Mampholo et al. [21] further reported a loss of chlorophyll, phenolic compounds, flavonoids, and total antioxidant activity in cabbage, Amaranthus, and nightshade stored at 10 °C. The loss of chlorophyll content and total antioxidant activity accompanied by a drastic reduction in moisture content, and soluble proteins was also reported in broccoli during storage at 20 °C [67]. These facilitated a loss of greenness and the texture of the broccoli, leading to poor appearance. Such findings suggest that the antioxidant activities are affected under higher and lower temperatures during the storage of leafy greens. Furthermore, there was a significant increase in the antioxidant enzymes, including PPO and PAL. This increment stimulated the accumulation of the total phenolic compounds and further increased the rate of browning [68]. Similar studies with high activities of antioxidant enzymes like PPO and POD were reported by Aguero et al. [69] in lettuce. The findings were also linked to the loss of greenness in lettuce, accompanied by the browning. In summary, the findings from the above studies revealed that quality deterioration in leafy vegetables is more biochemical, mainly because it is regulated by enzyme activities like chlorophyllase, which stimulate chlorophyll degradation, then the PPO and PAL facilitate the biosynthesis of the phenolic compounds, including flavonoids, and they also induce tissue browning.

In addition to the above traits, the quality deterioration of leafy vegetables has also been linked to the loss of ascorbic acid in various crops, including leeks, spinach, cabbage, Swiss chard, watercress, Amaranthus, and nightshade [21,66,70]. Agüero [69] reported a loss of ascorbic acid (AA) in lettuce during storage, which was linked to wilting. However, the authors revealed that the AA was not altered by the varying relative humidity, which influences the rate of moisture loss and wilting. Ambuko et al. [70] reported a positive correlation between wilting and a loss of AA in Amaranthus. Kader [71] reported contradicting results saying that a loss of AA was higher in the outer leaves of lettuce that are losing more moisture due to exposure to higher temperatures. This led to the concluding statement saying, that leafy vegetables lose AA during storage; however, it is not clear whether this is caused by high temperatures, low moisture, or both.

A loss of a number of biochemical constituents correlates with the loss of nutritional content. This was supported by the findings reported by Liao et al. [66], whereby a quality loss in cabbage, spinach, and leeks was linked to the loss of soluble proteins. Therefore, the above findings reveal that quality deterioration in leafy greens is linked to the number of enzymes associated with antioxidant activities. Degradation of the antioxidant activities triggered by antioxidant enzymes stimulates quality deterioration.

### 3.7. Hormonal Regulation of Leaf Senescence

**Figure 3:**
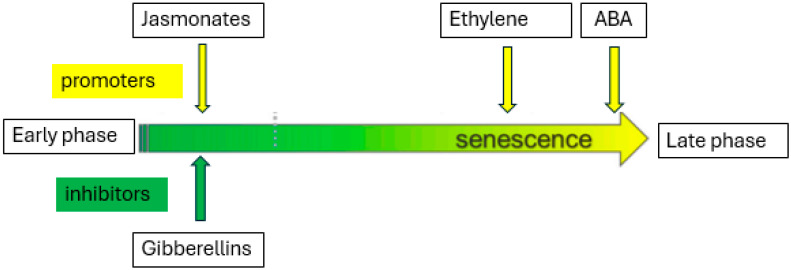
Hormonal effects in leaf senescence.

The senescence of leafy vegetables is also hormonally regulated (Figure 3). Just like in all other crops, the plant growth regulators are synthesized during the active growth period of leafy vegetables, and they are translocated into areas where they are actively functioning. Lipton [9] revealed that the senescence phase is closely linked to the levels and interactions of hormones, like cytokinin, gibberellin, ABA, and ethylene, and the water status. The plant hormones play different roles during preharvest and postharvest in leafy vegetables. Therefore, some hormones like jasmonates, ethylene, and abscisic acid (ABA) participate in inducing senescence in leafy greens [72]. This is associated with the concentrations of these hormones which increase as leaf tissues mature. While gibberellins inhibit the induction of senescence, as they become abundant at the early stages of leaf development [72].

**Table 2:** Physiological and biochemical response of leafy vegetables during storage.

Crop	Temperature (°C)	Physiological and/Biochemical Response During Storage	Reference
Lettuce	10	Loss of moisture, decrease in colour hue angle (h), loss of total chlorophyll, anthocyanin	[73]
Spinach			
Leek	4	Loss of soluble proteins, Vitamin C, Chlorophyll a	[66]
Cabbage			
Lettuce	0, 5, 10, 20	Loss of chlorophyll and colour hue angle (h). Increase in colour lightness (L)	[14]
Broccoli			
Watercress	−7, −15, −30	Loss of colour hue angle (h), chlorophyll, and ascorbic acid	[74]
		Reduction in POD activity	[74]
Swiss chard		Loss of ascorbic acid and chlorophyll content, no effect on peroxidase (POD)	[75]
Rocket Chicory	4 and 5	Loss of chlorophyll, anthocyanins, phenolic compounds, and carotenoids	[51]
Swiss chard			
Chinese Cabbage, Amaranthus Nightshade	10	Loss of ascorbic acid, carotenoids, total phenols, flavonoids, and antioxidant activity.	[21]
Broccoli	20	Drastically loss of moisture, total chlorophyll content, colour hue angle (h), and soluble proteins	[67]
Lettuce	4	Loss of chlorophyll content, antioxidant capacity, and colour hue. Increase in browning index, phenolic content, polyphenol oxidase (PPO), and phenylalanine ammonia (PAL)	[68]
Lettuce	2	Loss of moisture, chlorophyll content and ascorbic acid content	[69]
Lettuce	8	Loss of chlorophyll content, moisture, and increase in total phenolic content (TPC), PPO, and POD activity	[76]
Lettuce	4 and 8	Loss of chlorophyll and carotenoid contents. Increase in the respiration rate	[77]
Spinach			
Amaranth	4 and 10	Loss of moisture, colour hue angle (h), and vitamin C	[70]

## 4. Conclusions

The findings from the current study revealed that the tendency of fresh produce to be highly perishable is linked to high metabolic processes influenced by the storage environment. Respiration, ethylene production, moisture loss, chlorophyll degradation, and loss of texture are among the key attributes linked to quality deterioration. The mechanism of quality deterioration in leafy greens is not well explained in the literature; however, several studies indicated that it is mainly due to the loss of moisture since leafy vegetables consist of more than 80% moisture content. Quality deterioration of fresh greens is also linked to the accumulation of enzymes like chlorophyllase, which degrade chlorophyll, POD, PPO, and PAL, which participate in the biosynthesis of phenolic compounds, flavonoids, and other antioxidants. The direct relationship between the activities of these enzymes and quality deterioration in greens still needs to be explored in various fresh produce. The findings from this study revealed that a loss of quality in leafy vegetables further results in the loss of carotenoids, anthocyanins, vitamin C, and soluble proteins. This indicates a significant loss of dietary benefits associated with the consumption of leafy greens.

## 5. Future Prospects

Little has been reported on the loss of mineral nutrients during postharvest handling of fresh produce. Fresh greens are known for supplying a number of mineral nutrients including magnesium (Mg), potassium (K), iron (Fe), calcium (Ca), and zinc (Zn). Furthermore, greens are rich in vitamin K and dietary fibre. Elvira et al. [78] reported a loss of Mg and Ca in response to ozone treatment during the storage of Swiss chard at 16 °C. However, this study did not present a general trend of a change in mineral nutrients without postharvest preservative treatments. Therefore, it is recommended that future studies explore the status of mineral nutrients during postharvest storage of leafy vegetables. The interrelationship between the antioxidant activities and the mineral nutrients in leafy vegetables is also not well studied. The mineral nutrients serve as primary metabolites, while antioxidants serve as secondary metabolites; therefore, knowledge of the behaviour of both types of metabolites during the postharvest can aid in deducing their preservative measures.

## Figures and Tables

**Table 1:** Previous reviews on leafy vegetables with their main objectives and contribution to the body of knowledge.

Topic	Objective	Contribution	Reference
Degradation of chlorophyll during processing of green vegetables: A review	To explore different ways of retaining chlorophyll during the processing of leafy vegetables.	Mechanism of chlorophyll degradation was explained, and the models for predicting chlorophyll degradation were introduced.	[6]
Postharvest treatments of African leafy vegetables for food security in Kenya: A review	To review various studies on common postharvest handling and postharvest treatment of traditional ALVs grown and consumed in Kenya.	Revealed appropriate postharvest treatments of ALVs with emphasis on contributing to food security.	[7]
An overview of postharvest biology and technology of fruits and vegetables	To review the factors affecting deterioration of fruits and vegetables and the technologies that are used to preserve quality.	Discussed the factors affecting deterioration of fruits and vegetables and the technologies that are used to preserve their quality.	[8]
Neglected and Underutilized Cultivated Crops with Respect to Indigenous African Leafy Vegetables for Food and Nutrition Security	To explore the importance of the indigenous leafy vegetables and their importance in alleviating food insecurity worldwide.	The role of neglected and underutilized vegetable species, and indigenous African leafy vegetables in particular, is discussed with respect to their contribution to food and nutrition security.	[2]
Senescence of Leafy Vegetables	To understand the progress of senescence during storage of leafy vegetables.	Demonstrated that senescence is closely linked to the levels and interactions of hormones, like cytokinin, gibberellin, ABA, and ethylene, and the water status.	[9]
Biological and Biochemical Changes in Minimally Processed Refrigerated Fruits and Vegetables	To improve the like-fresh characteristics of fruits and vegetables to extend their shelf-life, thus allowing distribution within an area.	Demonstrated that proper processing and packaging minimize changes and quality loss with increased shelf-life.	[10]
Research Progress in Preservation of Postharvest Leafy Vegetables	To assess the research progress in preservation of postharvest leafy vegetables and shelf life.	The different postharvest technologies for preserving quality and extending shelf life of leafy vegetables were critically discussed.	[11]
Effect of Light-Emitting Diodes (LEDs) on the Quality of Fruits and Vegetables During Postharvest Period: A Review	To review the recent applications of LEDs in postharvest storage of fresh produce, including its effect on physiological characteristics, secondary metabolism, nutritional attributes, ripening process, senescence, shelf-life improvement, and pathogenic microbial spoilage of fruits and vegetables.	The application of LEDs holds the potential for life-enhancing storage and handling during postharvest activities, also can assist growers and vendors in reducing waste as well as aid in the provision of long-term storage and transportation.	[12]
Dietary phytonutrients in common green leafy vegetables and the significant role of processing techniques on spinach: A review	To provide a summary of the phytonutrients in such leafy greens with a detailed description of its bioavailability of nutrients, role of bio fortification, changes during harvest and postharvest processing.	Understanding the importance of the preharvest practices like fertilizer application aid in understanding the nutritional and dietary benefits of leafy vegetables.	[13]

## Data Availability

Not Applicable.
